# Assessing the Antimicrobial Effect of the Essential Oil of *Myrtus communis* on the Clinical Isolates of *Porphyromonas gingivalis*: An *in vitro* Study

**DOI:** 10.17795/jjnpp-12253

**Published:** 2013-11-01

**Authors:** Azita Hedayati, Hengameh Khosropanah, Abdollah Bazargani, Molud Abed, Amir Emami

**Affiliations:** 1 Department of Periodontics, Shiraz University of Medical Sciences, Shiraz, IR Iran; 2 Department of Bacteriology and Virology, Shiraz University of Medical Sciences, Shiraz, IR Iran

**Keywords:** *Porphyromonas gingivalis*, Periodontitis, *In vitro*

## Abstract

**Background:**

One of the major diseases affecting the oral health is periodontal disease. Various therapeutic methods have been introduced to eliminate the periodonto-pathic subgingival microflora. Among these, *Porphyromonas gingivalis* (*P. gingivalis*) has a major role in the pathogenesis of different forms of periodontal diseases.

**Objectives:**

The present study investigated the antimicrobial effect of the essential oil of *Myrtus communis* on *Porphyromonas gingivalis* (*P. gingivalis*) as the most destructive periodontal pathogens.

**Materials and Methods:**

The subjects included 27 male and 3 female patients with advanced chronic periodontitis. The mean age of the patients was 47.6 ± 2.0 years old. *P. gingivalis* was isolated from the samples and identified by various diagnostic tests, including Gram staining, Indol test, and fluorescent test. Minimum inhibitory concentration (MIC) of the essential oil against isolated *P. gingivalis* was determined by broth micro-dilution method.

**Results:**

In this study, 0.12 - 64 μL/mL *Myrtus communis* essence were used for 30 *P. gingivalis* isolates and the MIC50 and MIC90 concentration of *Myrtus communis* essence against the isolates was equal to 1 and 8 μL/mL respectively.

**Conclusions:**

The results showed that *Myrtus communis* has antimicrobial effects against *P. gingivalis*. Further studies are suggested to include this essence in therapeutic protocols of periodontal disease.

## 1. Background

The basic goal of dental treatment is to protect and maintain healthy tooth structures. One of the major diseases affecting the oral health is periodontal diseases. The symptoms of the disease start with inflammation of the gingival tissue in the form of gingivitis and continue to involve supporting structures around the teeth in the form of periodontitis (1). Inflammation is a double-edged blade; on one side, it causes damage to tissues and on the other side it stimulates cells to restore lost structures. The accumulation of the bacterial plaque starts the periodontal disease, and host-defense responses follows the process of the disease (2). Various therapeutic methods including surgical and non-surgical techniques have been introduced to eliminate the periodontopathic subgingival microflora (3). Among these, *Porphyromonas Gingivalis* (*P. gingivalis*) has a major role in the pathogenesis of different forms of periodontal diseases such as chronic and refractory periodontitis, as well as periodontal and pericoronal abscess (4-6).

Numerous factors are engaged in the pathogenesis of *P. gingivalis* including collagenase activity, fibroblast inhibition, invasion to epithelial cells which results in widening of the junctional epithelium and penetration of microorganism in to the connective tissue (7). The presence of endotoxins and the cytokine secretion of *P. gingivalis* induce bone resorption. In addition, these microorganisms have the ability to inhibit immunoglobulins and IL-8 secretion by production of proteolytic enzymes (8, 9). *Myrtus* is a genus of one or two species of flowering plants in the Myrtaceae family (10). It is a shrub form plant with dark green leaves, large flowers and small bluish-black fruits. There are some protuberances on its stem called Galle. All parts can be used for different therapeutic purposes. It has been shown that the crude and methanol extract of *M. communis* depicts antimicrobial activity on the growth inhibition of some bacteria, such as Gram positive *Streptococcus agalactiae* and *Listeria monocytogenes* as well as gram negative bacteria (11). Also, in vitro and in vivo experiments proved that some *M. communis* leaves contain materials that have the potential to inhibit the biosynthesis of eicosanoids by direct inhibition of cyclooxygenase-1 and 5-lipoxygenase. In fact, their capability in suppressing the typical pro-inflammatory cellular responses obscured their benefit in treatment of the inflammatory and allergy-related diseases (10). Although *Myrtus communis* may have shown to be a potential agent against several bacteria, no data are available on the *P. gingivalis*.

## 2. Objectives

The present in vitro study aims to assess the antimicrobial effect of *Myrtus communis* on the most periodonto-pathogen species; i.e. *P. gingivalis*.

## 3. Materials and Methods

### 3.1. Patient Selection

Thirty patients with advanced chronic periodontitis who were referred to the department of periodontology Shiraz dental school between October 2010 and June 2011 were enrolled into the study. The participants had at least one tooth with pocket deeper than 7 mm in the initial examinations. The patients, who had a history of periodontal treatment, smoking, pregnancy, taking antibiotics during the past two months, systemic disorders such as diabetes and infectious diseases, taking iron supplements were excluded from the study. The recruited cases should have been at least 35 years old. The age of the selected patients ranged from 36 to 52 years old. Twenty seven of these patients were male, while three subjects were female.

The extra oral examinations was performed and a complete medical history was obtained .The chief complaint was recorded and Pocket depth (PD) and mucogingival junction (MGJ) was measured. The gingiva was examined and the type of the periodontal disease was identified. The patients were then instructed for oral hygiene using soft toothbrush (Trisa ultra supersensitive) and unwaxed dental floss (oral-B). All the patients underwent supragingival scaling using Hu-friedy ultrasonic device and hand instruments. One week after the supragingival scaling, the patients were called for sampling. The samples were taken from all the areas which had more than 7 mm pocket depth, by a single stroke of a curette on the tooth surface and insertion of a No.20 paper cone in the periodontal pocket for 15, 20 and 30 seconds. The specimens were placed in 5 mL test tubes containing 3 mL Thioglycolate culture medium (Merck) and were kept in the refrigerator at 4˚C temperature. To avoid the interference at the moment of the withdrawal of the curette or the paper cone, teeth with crowns or subgingival filling were also excluded. The tubes were then put in special molds to be prevented from blending and transferred to the laboratory in less than 20 minutes.

### 3.2. Media 

#### 3.2.1. *Brucella* Broth Medium

From the culture medium (Merck), 41 g was dissolved in distilled water and sterilized for 15 minutes in an autoclave. Then under aseptic conditions, 5% lysed sheep blood, 5 μg/mL Hemin, 1 μg/mL vitamin K, 1 μg/mL nalidixic acid, and 5 μg/mL vancomycin were added to the sterile medium.

#### 3.2.2. Brucella Blood Agar Medium

The same previous procedure was was repeated and 1.5% agar powder (Merck) was added and sterilized in an autoclave for 15 minutes. Afterwards, under aseptic conditions, 5% lysed sheep blood, 5μg/mL Hemin, 1 μL/mg vitamin K, 1 μg/mL nalidixic acid, and 5 μg/mL Vancomycin were added to the sterile medium.

### 3.3. Bacterial Identification

The prepared samples were inoculated on *Brucella* blood agar medium and incubated under anaerobic conditions at 37˚C for 14 days. Subsequently, the dark brown colonies grown on the medium were picked up and investigated by gram staining and fluorescent light. The bacteria with black or dark brown pigments (g^-^) and fluorescent (-) were identified as *P. gingivalis*. To confirm the identification, Indol test was employed on these bacteria and samples with positive results were utilized in the experiment. After the isolation and the identification of the bacteria, they were transferred to the cooked meat medium for higher proliferation and incubated at 37˚C for 14 days in an anaerobic jar.

### 3.4. Preparation of Essential Oil 

First, 300 g of *Myrtus communis* powder (powder form of plant increase the yield effectively) immersed in distilled water for 4 hours, which is then boiled in a Clevenger (yield 0.69%). The essence was obtained then for more purification the additional moisture was removed by sodium sulfate powder and stored at 4˚C until use. The ״purification״ in this method means removal of excess water. The material used in this study was kindly offered by the center of Medicinal and Herbal Chemistry Research center by professor Javidnia.

### 3.5. Broth Microdilution Method

In this study, broth micro dilution method was used in order to determine the Minimum Inhibitory Concentration (MIC) of *Myrtus communis* essence on *P. gingivalis*. In this method, 96-well cell cultured micro plates with flat-bottom U-shaped wells were used. First, under sterile conditions and under a laminar hood, 100 μL of the *Brucella* broth medium were added to each row of wells of the first, 3rd and up to 12th column. After adding 128 μL of *Myrtus communis* essence to the second row of wells, their volume was increased to 200 μL using the *Brucella* broth medium so the essence dilution reached 64 μL/mL. Then, 100 μL was taken from the second well and added to the third one. This trend was continued up to the 11th well and finally, 100 μL of the 11th well was thrown away. Serial dilution was prepared from the investigated compounds in the 2nd-11th columns wells. Then the 0.5 McFarland suspension (1 × 10^8^ CFU/mL) of tested bacteria was diluted 1:10 to yield 10^7^ CFU/mL. Total of 5 μL of this suspension was inoculated into to the 2 nd-12 th columns’ wells, in this way the final test concentration of bacteria in each well was approximately 5 × 10^5^ CFU/mL (or 5 × 10^4^ CFU/well). To control the sterility of the conditions; the well of the first column which contained 100 μL of the culture medium without the microbial suspension were considered as negative control. On the other hand, the well of the 12^th^ column which included the microbial suspension and lacked *Myrtus communis* essence were considered as positive control. Then, the plates were placed under anaerobic conditions at 37˚C. The turbidity of micro plate wells which contained bacteria were read by naked eyes after 72 hours. The concentration of *Myrtus communis* essence in the first well in each column which had no turbidity was considered as MIC. In order to perform the mentioned experiments, the bacteria had to be in the logarithmic phase. Thus, the fresh bacterial culture which was kept in cooked meat medium at 37˚C for 4 days under anaerobic atmosphere was utilized. All the experiments were repeated for 3 times in order to decrease the error rate to the possible extent. Also, in case of absence of matching among the rows, the experiments were repeated again (12).

## 4. Results

The subjects of the present study included 27 male and 3 female patients with advanced chronic periodontitis. The mean age of the patients was 47.6 ± 2.0 years old. Sampling was performed and *P. gingivalis* was isolated from the samples through various experiments, including gram staining, Indol test, and fluorescent test. In this study, 0.12 - 64 μL/mL *Myrtus communis* essence were used for 30 *P. gingivalis* isolates and the MIC50 and MIC90 concentration of *Myrtus communis* essence against the isolates were equal to 1 and 8 μL/mL, respectively.

## 5. Discussion

The present *in vitro* study showed that 1 μL/mL concentration of *Myrtus communis* essence had an inhibitory potential against *P. gingivalis* as the most periodonto-pathogen species. Chronic periodontitis is known as an infectious disease which is triggered and maintained by microbial plaque, but further tissue damage can result from host- defense mechanisms (1).

The goal of most medical or dental treatments is to remove the disease initiating factor. Among the dental plaque microorganisms, *P. gingivalis* is a predominant gram-negative anaerobic periodontal pathogen (13). The usual treatment for bacterial elimination or reduction is mechanical debridement of the root surfaces, but it is not effective for the pathogens that have penetrated the tissues (14). Therefore, the adjunctive techniques such as utilization of antibiotics were considered (15). The use of systemic antibiotics has the potential of unwanted side effects such as gastrointestinal disturbances, allergic reactions, drug resistance and lack of cooperation from the patients (16). Topically used antibiotics have fewer side effects than systemic antibiotics. The most important benefit of them is their favorable effect with the minimum dose of administration. But the disadvantages of topical antibiotics are time-consuming, high cost and technique sensitivity (17). Another method is to use different agents in the form of mouthwashes. Chlorhexidine is the most useful mouthwash, but because of tooth and tongue staining and its unpleasant taste, it is not acceptable for the patients (18). Listerine is another mouthwash, which although it does not have the side effects of chlorhexidine, it has high levels of alcohol that results in mouth dryness and subsequent complications (19). Most patients prefer natural plants rather than synthetic medications. *Myrtus communis* is one of them. Aerial Branches of the plant were used in this study. One of the preparations of *Myrtus communis* has been shown to have an inhibitory effect on gram-positive aerobic micro-organisms such as *staphylococcus aureus*, *E. coli* and *pseudomonas aeruginosa* (20). Topical of this herbal medication can be used in the treatment of herpes simplex and nasal inflammation. Rinsing of this herbal medicine is effective in the treatment of aphtous ulcer (10). In this study, we used clinical strains of *P. gingivalis* isolated from the patients instead of standard strains. Because the reference strains were isolated a long time ago (several years) and the frequent cultures and subsequent mutations might have rendered them different from the strains which exist in human societies now a days. Thus, they might not be appropriate to investigate their resistance against pharmaceutical compounds.

We evaluated the antimicrobial effect of *Myrtus communis* against the most periodonto-pathogen species; i.e. *P. gingivalis* which is a gram negative bacterium. The concentration range of *Myrtus communis* against *P. gingivalis* was between 0.12 and 64 μL/mL and the MIC50 on the isolated P. gingivalis was 1 μL/mL. These results are in agreement with the study conducted by Sulieman et al. who used this medicine as a root canal irrigant (20). The findings of his study indicated that the alcoholic extract of *Myrtus communis* showed antimicrobial effects at different dilutions, but the best antibacterial effect was noticed at the dilution of 35%. The strength of this study is the utilization of distilled water extract of the leaves rather than its alcoholic extract, which represents the pure effect of the plant. We did not determine the composition of the essence in this study. This essential oil is cheap and easily prepared without any unwanted effects. Moreover, it has a good smell which would positively influence its acceptance among the patients. The current study was the only one investigating the effect of *Myrtus communis* essence on *P. gingivalis* anaerobic bacterium. This essence is easily prepared, low-priced, with a delicate smell with no adverse effects. Further studies are recommended on the clinical effects of this essence, as a mouthwash, toothpaste, or subgingival irrigant; alone or in combination with scaling and root planning treatment.

## References

[1] Flemmig TF (1999). Periodontitis.. Ann Periodontol..

[2] Page RC (1991). The role of inflammatory mediators in the pathogenesis of periodontal disease.. J Periodontal Res..

[3] Haffajee AD, Teles RP, Socransky SS (2006). The effect of periodontal therapy on the composition of the subgingival microbiota.. Periodontol 2000..

[4] Kumar PS, Griffen AL, Barton JA, Paster BJ, Moeschberger ML, Leys EJ (2003). New bacterial species associated with chronic periodontitis.. J Dent Res..

[5] Haffajee AD, Socransky SS, Dzink JL, Taubman MA, Ebersole JL (1988). Clinical, microbiological and immunological features of subjects with refractory periodontal diseases.. J Clin Periodontol..

[6] Herrera D, Roldan S, Sanz M (2000). The periodontal abscess: a review.. J Clin Periodontol..

[7] Zambon JJ (1996). Periodontal diseases: microbial factors.. Ann Periodontol..

[8] Huang GT, Haake SK, Kim JW, Park NH (1998). Differential expression of interleukin-8 and intercellular adhesion molecule-1 by human gingival epithelial cells in response to Actinobacillus actinomycetemcomitans or Porphyromonas gingivalis infection.. Oral Microbiol Immunol..

[9] Darveau RP, Belton CM, Reife RA, Lamont RJ (1998). Local chemokine paralysis, a novel pathogenic mechanism for Porphyromonas gingivalis.. Infect Immun..

[10] Chevallier A (1996). The encyclopedia of medicinal plants..

[11] Mansouri S, Foroumadi A, Ghaneie T, Gholamhosseinian Najar A (2001). Antibacterial activity of crude extracts and fractionated constituents of Myrthus Communis.. Pharm Biol..

[12] Tamashiro L, Isenberg HD (1995). Preparation of broth microdilution MIC tray.. Clinical microbiology procedures..

[13] Papapanou PN, Neiderud AM, Papadimitriou A, Sandros J, Dahlen G (2000). "Checkerboard" assessments of periodontal microbiota and serum antibody responses: a case-control study.. J Periodontol..

[14] Greenstein G (1994). Re: A review of longitudinal studies that compared periodontal therapies (J Periodontol 1993;64:243-253).. J Periodontol..

[15] van Winkelhoff AJ, Rams TE, Slots J (1996). Systemic antibiotic therapy in periodontics.. Periodontol 2000..

[16] Herrera D, Sanz M, Jepsen S, Needleman I, Roldan S (2002). A systematic review on the effect of systemic antimicrobials as an adjunct to scaling and root planing in periodontitis patients.. J Clin Periodontol..

[17] Mombelli A, Samaranayake LP (2004). Topical and systemic antibiotics in the management of periodontal diseases.. Int Dent J..

[18] McCoy LC, Wehler CJ, Rich SE, Garcia RI, Miller DR, Jones JA (2008). Adverse events associated with chlorhexidine use: results from the Department of Veterans Affairs Dental Diabetes Study.. J Am Dent Assoc..

[19] McCullough MJ, Farah CS (2008). The role of alcohol in oral carcinogenesis with particular reference to alcohol-containing mouthwashes.. Aust Dent J..

[20] Suliman RT (2009). The Antibacterial Effect of Myrtus communis as Root Canal Irrigant: A comparative Study.. Al–Rafidain Dent J..

